# Avoiding Deception and Nondisclosure in Clinical Practice: A Narrative Review of Ethical Principles, Legal Perspectives, and Communication Strategies

**DOI:** 10.7759/cureus.92424

**Published:** 2025-09-16

**Authors:** Kasidid Lawongsa

**Affiliations:** 1 Family Medicine, Phramongkutklao Hospital, Bangkok, THA

**Keywords:** deception in healthcare, informed consent, medical ethics, nondisclosure, physician–patient relationship

## Abstract

Deception and nondisclosure in clinical practice undermine patient autonomy, informed consent, and trust in the physician-patient relationship. This review synthesizes ethical principles, legal frameworks, and communication strategies to address these issues, drawing on national and international guidelines. Deception, including lying, misrepresentation, and withholding critical facts, violates the principles of honesty, respect for autonomy, nonmaleficence, and justice, with significant implications for patient safety and public confidence. Factors contributing to such practices include therapeutic privilege, epistemic uncertainty, cultural norms, institutional pressures, and fear of litigation. Legal and professional standards, such as those from the Medical Council of Thailand, the American Medical Association, and the UK General Medical Council, mandate truthful and timely disclosure. Evidence-based communication approaches, including structured protocols like SPIKES, plain language use, and gradual disclosure, can help clinicians convey sensitive information while minimizing distress. Institutional measures, such as formal error disclosure policies, ethics training, and emotional support systems, are critical in fostering a culture of transparency. Sustained commitment to these principles is essential for upholding professional integrity, enhancing patient trust, and improving healthcare outcomes. This is a narrative review based on targeted searches of PubMed, Scopus, and Google Scholar, as well as professional guidelines and regulatory documents.

## Introduction and background

Breaches of confidentiality are among the most common ethical missteps in clinical practice. They occur when sensitive patient information is disclosed or mishandled without proper consent. Sometimes these incidents arise from carelessness or weak safeguards, but whatever the cause, they inevitably strain the physician-patient relationship and weaken professional accountability [1,2].

At the other end of the spectrum lies medical fraud. Fraud goes beyond accident or oversight; it involves the deliberate use of deception, concealment, or misrepresentation, usually with the awareness that such actions may lead to financial gain or personal advantage. Unlike an error, fraud reflects a conscious choice to mislead, and its effects ripple outward, harming patients, damaging institutions, and undermining public confidence in health systems [1,2].

Yet the divide between “error” and “fraud” does not capture every form of deception. A further category is deliberate misrepresentation or selective nondisclosure that is not tied to financial benefit but rather to sustaining a particular policy or preserving institutional authority. The controversies over COVID-19 responses illustrate this complexity. Some critics argue that the limited evidence base for lockdowns or vaccine policy was not fully acknowledged by public authorities or clinicians. The intent may not have been enrichment but rather reassurance and compliance. Even so, these actions involved distortion and omission, which risk eroding trust, narrowing scientific debate, and limiting the public’s ability to make informed choices.

In contrast, classic examples of fraud, such as false advertising of health products, billing for services not provided, falsifying records, or promoting unproven treatments, clearly involve purposeful intent to mislead [1-3].

Globally, the scale of fraud is immense. In the United States, healthcare fraud and improper payments are estimated to exceed US$100 billion annually [2,4]. In FY 2024, the Centers for Medicare & Medicaid Services reported US$87.1 billion in improper payments across Medicare, Medicaid, CHIP, and the Premium Tax Credit Exchange [5]. In 2025, a national fraud takedown led to charges against 324 defendants in schemes exceeding US$14.6 billion [6-8]. In Thailand, though the problem is less well quantified, cases of misleading advertising, fraudulent health product promotion, and inappropriate billing have been reported in both the public and private sectors. Such practices threaten patient safety and corrode public trust in the profession.

This review examines medical fraud from the perspectives of ethics, law, and prevention. We highlight its novelty as a multifaceted problem that operates across individual, institutional, and systemic levels. Fraud may involve not only individual practitioners but also administrators, insurers, corporations, and even regulators, each with distinct motives. By combining ethical theory with practical examples, we aim to provide a framework that promotes accountability, transparency, and trust, foundational elements of high-quality healthcare.

This review aims to synthesize ethical, legal, and preventive perspectives on deception and medical fraud, with particular emphasis on physician-patient communication, institutional responsibilities, and strategies to promote transparency and trust.

This paper is a narrative review. Literature was identified through targeted searches of PubMed, Scopus, and Google Scholar between January 1980 and June 2025, using combinations of keywords and MeSH terms such as “medical fraud”, “clinical deception”, “nondisclosure”, “informed consent”, “medical ethics”, “duty of candor”, and “healthcare advertising”. Sources were included if they were peer-reviewed publications, professional guidelines, or regulatory documents addressing ethical, legal, or preventive aspects of deception or fraud in healthcare communication, and both English- and Thai-language materials were considered. We excluded non-healthcare fraud literature (e.g., general criminal or financial fraud outside medical contexts), duplicate publications, and opinion pieces without empirical or regulatory grounding. Titles and abstracts were screened for relevance, with full texts reviewed where necessary. Although formal appraisal instruments (such as Cochrane risk of bias, ROBIS, or CASP checklists) were not applied due to the narrative nature of the review, all sources were evaluated for credibility and conceptual contribution. Findings were narratively synthesized with emphasis on conceptual breadth and illustrative case examples rather than quantitative pooling or meta-analysis.

## Review

Definitions and conceptual framework

Deception in medical practice can take different forms and should be distinguished from fraud. In the clinical context, deception refers to communication practices in which information is presented in a way that may mislead patients or families, for example, by withholding details of a terminal prognosis to maintain hope. This form of deception arises primarily in the domain of physician-patient communication and is often debated within the framework of medical ethics, focusing on autonomy, beneficence, and cultural values.

By contrast, medical fraud involves deliberate misrepresentation or concealment for personal or institutional gain, such as falsifying records or submitting false claims for reimbursement. Because the motivations, consequences, and regulatory implications differ, these two phenomena require separate analytical frameworks: one rooted in clinical ethics and communication, and the other in law, accountability, and professional integrity [1-3].

This can include deliberate falsehoods, exaggerations, selective omission of critical facts, or presenting information in a misleading manner [9]. Deception may occur in clinical encounters, research settings, or public communication of medical information, and it represents a breach of both ethical and professional duties. In its various forms, deception undermines the fundamental expectation that physicians act as trustworthy stewards of patient welfare.

Nondisclosure is the intentional withholding of important information that could reasonably influence a patient’s decision-making regarding their diagnosis, prognosis, or treatment [10]. Unlike direct deception, nondisclosure does not necessarily involve the communication of false information but rather the failure to disclose facts that are material to the patient’s health-related choices. Examples include withholding a poor prognosis, omitting discussion of available treatment alternatives, or failing to disclose known risks of a procedure. Although physicians sometimes justify nondisclosure as a means to reduce distress or maintain hope, ethical scholarship emphasizes that such omissions compromise patient autonomy, weaken informed decision-making, and may violate legal standards governing consent.

A clear distinction can be made between lying, misrepresentation, and withholding information [11]. “Lying” refers to providing a statement that is knowingly false, with the intent to deceive. Misrepresentation occurs when information is presented in a way that creates a false impression, even if certain elements are factually correct, for example, overstating the benefits of a treatment while minimizing its risks. Withholding information involves the failure to disclose relevant facts that a patient has the right to know, thereby limiting their ability to make an informed decision. Although these practices differ in form, they share the common ethical problem of undermining honesty, autonomy, and the fiduciary responsibility inherent in the physician-patient relationship.

These concepts are closely linked to the ethical principles of truth-telling and informed consent. Truth-telling obligates healthcare professionals to be honest and transparent with patients, thereby fostering trust and respecting the patient’s moral right to self-determination [12]. Informed consent, as defined in bioethics and law, requires that patients receive adequate, accurate, and comprehensible information about their medical condition, available options, and associated risks and benefits, enabling them to make voluntary and informed choices [13]. Both deception and nondisclosure undermine informed consent by restricting the patient’s access to relevant information, thereby compromising autonomy and exposing patients to potential harm.

In addition, the conceptual framework emphasizes that deception and nondisclosure must be evaluated not only in terms of individual physician behavior but also at the institutional and systemic levels. History demonstrates that public authorities and healthcare institutions can engage in active deception, not merely by fostering problematic cultures but through deliberate concealment or distortion of information. Examples include the thalidomide tragedy, the decades-long concealment of risks in the UK contaminated blood scandal, and, more recently, controversies surrounding the transparency of COVID-19 policies. While individual-level safeguards such as professional codes of conduct, appraisal and revalidation systems, and the oversight of professional regulators (e.g., the General Medical Council) are well established, institutional-level controls remain less clearly defined. Effective oversight at this level should include independent regulatory scrutiny, transparent public reporting of adverse outcomes, robust whistleblower protections, and external auditing mechanisms. Embedding such measures alongside individual-level safeguards is essential to ensure accountability, prevent systemic deception, and sustain public trust in medicine.

Equally important, deception and nondisclosure must also be understood within wider systemic and cultural contexts. Institutional pressures, such as fear of litigation or reputational damage, can influence disclosure practices, while cultural norms may shape whether physicians prioritize family interests or patient autonomy in truth-telling. Thus, a comprehensive understanding of deception and nondisclosure requires attention not only to individual ethics and professional codes but also to the broader social and cultural frameworks that govern medical practice [9-13].

Ethical considerations

Ethical evaluation of deception and nondisclosure must be grounded in the four core principles: autonomy, beneficence, nonmaleficence, and justice. Breach of confidentiality clearly violates respect for autonomy and trust, but other forms of deception can also be analyzed through this lens. For example, during the COVID-19 pandemic, questions arose about whether public authorities and institutions fully disclosed the limitations of evidence regarding lockdowns and vaccine policy. From an ethical standpoint, selective nondisclosure in this context undermines autonomy by restricting the public’s ability to make informed decisions, may conflict with beneficence and nonmaleficence if policies cause unintended harms, and raises issues of justice when burdens and risks are unevenly distributed across populations. This example illustrates that ethical concerns about deception extend beyond individual physician-patient encounters to encompass the responsibility of institutions and governments to communicate transparently, even under conditions of uncertainty.

Several core ethical principles are directly relevant to avoiding deception and nondisclosure in medical practice. Honesty entails a moral duty for physicians to provide truthful, accurate, and complete information to patients, forming the foundation of professional integrity and protecting patients from misinformation and harm [14]. Honesty in medical practice goes beyond avoiding deliberate falsehoods; it requires active transparency in discussing diagnoses, prognoses, treatment options, and risks. Without honesty, the physician-patient relationship risks devolving into paternalism, where decisions are made on behalf of patients without their fully informed input.

Respect for autonomy recognizes patients’ rights to make their own healthcare decisions based on adequate, accurate, and comprehensible information [15]. This principle emphasizes that patients are the ultimate decision-makers regarding their own health, and their choices must be grounded in a clear understanding of their situation. Any form of deception or withholding of critical facts compromises informed consent and undermines this autonomy. For example, selectively omitting details of side effects or overstating treatment benefits interferes with the patient’s ability to exercise self-determination.

Nonmaleficence, the obligation to “do no harm,” extends beyond physical safety to include avoiding harm through misinformation, exaggeration, or omission [16]. Deceptive communication can result in inappropriate treatment choices, unnecessary interventions, or delays in receiving effective therapy. Psychological harm may also occur when patients later discover that they were misled, leading to feelings of betrayal, anxiety, or loss of control. Thus, harm is not limited to tangible medical outcomes but also encompasses emotional and moral injury.

Justice demands fairness in healthcare, ensuring that all patients have equal access to truthful information, and it requires that deceptive practices are not tolerated and that particular care is taken to ensure vulnerable individuals or groups are not disproportionately harmed or disadvantaged [17]. For example, selectively providing complete information only to more educated or financially privileged patients, while withholding it from vulnerable populations, constitutes an injustice. Justice requires that standards of truth-telling and disclosure be applied consistently across all patient interactions, regardless of socioeconomic or cultural background.

Relationship to trust in the physician-patient relationship

Trust is the cornerstone of effective medical care. It enables patients to share sensitive information, follow treatment plans, and engage meaningfully in shared decision-making [18]. Trust is built gradually through consistent honesty, transparency, and respect, but it can be quickly eroded by a single instance of deception or nondisclosure. Breaches of honesty damage the therapeutic alliance and can have long-term consequences for patient outcomes, compliance, and the reputation of the medical profession.

Once trust is broken, it can be extremely difficult to rebuild. This principle applies not only to the physician-patient relationship but also to the wider institutional and societal context. Public trust in the medical profession, healthcare institutions, and governmental health authorities has been eroded by repeated scandals, including the mishandling of the COVID-19 response. In many cases, the absence of an acknowledgement of wrongdoing or a meaningful apology has further compounded the loss of confidence. Without transparency and accountability, efforts to restore trust at either the individual or institutional level are unlikely to succeed. Restoring trust often requires sustained transparency, acknowledgment of wrongdoing, a genuine apology, and demonstrable changes in behavior [19]. However, even with these efforts, some patients may never fully regain confidence in their physician, highlighting the profound ethical and relational stakes involved in truth-telling.

Illustrative examples of ethical breaches

Examples of ethical breaches in clinical practice clearly illustrate the potential harm caused by deception and nondisclosure. One common instance is exaggerated advertising, where a private clinic may claim a “100% cure rate” for a cosmetic or elective procedure without peer-reviewed evidence. Such misrepresentation violates the principle of honesty, exploits patient vulnerability, and can drive individuals toward unnecessary or even risky interventions. Another breach involves withholding critical information, such as when a physician fails to disclose a known complication risk of a surgical procedure. By omitting this information, the physician deprives the patient of the opportunity to weigh benefits against potential harms, thereby invalidating informed consent and exposing the patient to unacknowledged risks. A further example is the promotion of unsupported therapies, in which a healthcare provider endorses an unproven herbal supplement for cancer treatment. Patients misled by such claims may delay or decline evidence-based therapies, resulting in disease progression and preventable morbidity or mortality. Collectively, these examples highlight how breaches in honesty and transparency can undermine patient autonomy, compromise safety, and erode trust in the medical profession.

Even well-intentioned forms of deception, such as withholding information to “maintain hope,” can have harmful consequences when they limit patients’ ability to make informed, autonomous choices.

Factors influencing deception and nondisclosure

Deceptive practices and withholding of information in healthcare can occur for various reasons, often influenced by a complex interplay of individual, cultural, and systemic factors. Recognizing these influences is essential for developing effective interventions that promote transparency, uphold ethical principles, and strengthen the physician-patient relationship.

Therapeutic Deception

Some physicians may withhold or modify information out of concern that full disclosure could cause emotional distress, loss of hope, or treatment refusal by the patient [20]. This is sometimes referred to as therapeutic privilege, in which the physician justifies nondisclosure on the grounds of protecting the patient’s well-being. Physicians may believe that revealing a grim prognosis could worsen anxiety, depression, or adherence to treatment. While such practices may be well-intentioned, they risk slipping into paternalism by substituting the physician’s judgment for the patient’s right to know. Ethical frameworks stress that therapeutic privilege should only be invoked under exceptional circumstances and always with careful consideration, documentation, and efforts to revisit disclosure when appropriate. Overuse of this justification undermines autonomy, weakens trust, and may expose physicians to legal liability.

Epistemic Uncertainty

Uncertainty in diagnosis, prognosis, or treatment outcomes (epistemic uncertainty) is another factor influencing nondisclosure [21]. Physicians may delay or limit communication until more definitive test results or specialist opinions are available. While caution in communication is sometimes necessary to prevent unnecessary alarm, a complete withholding of preliminary findings can deprive patients of the chance to prepare for potential outcomes or seek second opinions. For example, not disclosing a suspicious imaging result until confirmation is obtained may reduce immediate anxiety, but it also prevents the timely involvement of the patient in decisions about further investigations. Best practice suggests that physicians should acknowledge uncertainty openly, framing it as part of the medical process, which can actually foster trust rather than diminish it.

Cultural Norms and Family-Centered Disclosure

Cultural values strongly shape expectations of truth-telling in healthcare. In some societies, it is customary for healthcare professionals to inform the patient’s family before the patient or even to avoid direct disclosure to the patient altogether [22]. This family-centered disclosure model may arise from cultural notions of collectivism, where the family is regarded as the primary decision-maker and protector of the patient. While cultural sensitivity is important, it must be balanced with legal requirements and ethical obligations to respect individual autonomy. Challenges emerge when family requests for nondisclosure conflict with the patient’s right to informed consent. Physicians must navigate these situations carefully, employing culturally competent communication strategies while ensuring that the patient’s voice remains central in decision-making whenever possible.

Institutional or Systemic Pressures

Organizational and systemic factors also influence transparency. Healthcare institutions may discourage open disclosure of adverse events due to concerns about reputation, financial liability, or regulatory repercussions [23]. For example, hospitals may adopt risk-management strategies that prioritize damage control over patient disclosure, leading to selective communication or minimization of errors. Commercial interests can further distort information-sharing, such as in the case of pharmaceutical or device promotions that emphasize benefits while downplaying risks. Beyond these settings, regulators and advisory bodies, including the FDA, the UK Medicines and Healthcare Products Regulatory Agency, the General Medical Council (GMC), national departments of health, and organizations such as NICE or SAGE, also play a critical role in shaping transparency and disclosure.

However, history has shown that even within such formal structures, pressures of groupthink, institutional loyalty, or political influence can foster collusive behaviors and limit open debate. While policies, guidelines, and reporting systems form an essential foundation for accountability, they are not sufficient on their own. Combating these challenges requires additional safeguards, including independent oversight, strong protections for whistleblowers, and organizational cultures that value dissent and open scientific dialogue. Without such measures, ethical standards are compromised, and public trust in healthcare systems is eroded.

Fear of Litigation

Fear of malpractice lawsuits remains a significant barrier to full disclosure of medical errors [24]. Physicians may fear professional punishment, reputational harm, or financial penalties, leading them to practice defensive medicine or avoid acknowledging mistakes altogether. However, evidence suggests that concealment often increases litigation risk when patients discover the truth through other means. Conversely, open disclosure, accompanied by apology and corrective action, can reduce the likelihood of lawsuits and foster reconciliation. Encouraging such practices requires institutional commitment, including training programs in communication skills, mentorship schemes where experienced clinicians model transparent disclosure, and access to dedicated support services for clinicians under stress. These measures not only strengthen individual confidence in handling difficult conversations but also embed openness and accountability into organizational culture. A “just culture” approach, where errors are openly discussed, systemic contributors are addressed, and punitive measures are minimized, can help alleviate these fears. Such frameworks encourage learning from mistakes while maintaining accountability, striking a balance between patient rights and professional protection.

Summary

Taken together, these factors, ranging from physician intentions and epistemic uncertainty to cultural norms, institutional pressures, and legal concerns, highlight the multifaceted challenges surrounding deception and nondisclosure in medicine. Addressing them requires multi-level strategies: education in ethical reasoning, culturally competent communication, supportive institutional policies, and legal reforms that promote openness while safeguarding both patients and providers.

Legal and professional standards

Medical professionals are bound by ethical codes, statutory regulations, and institutional policies that emphasize truthfulness, transparency, and the avoidance of deceptive practices. These frameworks serve to protect patient rights, maintain professional integrity, and ensure public trust in the healthcare system.

Thai Medical Council Regulations

The Medical Council of Thailand, operating under the Medical Profession Act B.E. 2525 (1982), has issued regulations and ethical codes on the maintenance of medical ethics. These frameworks emphasize the physician’s duty to provide patients with accurate, clear, and sufficient information regarding their diagnosis, treatment options, associated risks, and prognosis. They also prohibit false, exaggerated, or misleading statements, particularly in the advertising of medical services or products, and call for professionalism in all communications, including those delivered through public media and social platforms. While the authoritative texts are issued in Thai, English-language summaries consistently highlight these principles, underscoring their central role in guiding professional conduct [25].

International Ethical Guidelines

American Medical Association (AMA) Code of Medical Ethics: The AMA Code states that physicians have a duty to disclose relevant medical information to patients in a timely and understandable manner. Ethical Opinion 8.12 (“Patient Information”) emphasizes that withholding information without the patient’s consent, except in rare circumstances of imminent harm, violates the principles of respect for autonomy and honesty [26].

GMC, UK: The GMC’s Good Medical Practice guidance mandates openness and candor. Paragraphs 68-71 state that doctors must be honest when things go wrong, promptly inform patients about adverse events, explain what happened, offer an apology, and set out actions to prevent recurrence [27].

Clinical contexts and case examples

Deception and nondisclosure can manifest in a variety of clinical and professional settings, from bedside care to institutional communication and public health messaging. These examples highlight the real-world consequences of breaches in honesty and underscore how adherence to ethical principles can strengthen patient safety, autonomy, and trust.

Medical Error Disclosure

One of the most critical contexts where honesty is tested is in the disclosure of medical errors. For instance, when a surgical patient is found to have a retained gauze swab in the abdominal cavity after an operation, there may be a temptation to minimize or delay disclosure out of fear of litigation or reputational damage. However, the principles of transparency and honesty require that this error be disclosed promptly and directly to the patient and their family [25,27].

When a medical error occurs, best practice requires that the patient be informed about the incident clearly and without concealment, ensuring that the communication is transparent and honest. Physicians should explain the clinical implications, including any potential risks or complications that may result, so the patient has an accurate understanding of the situation. It is also essential to outline the corrective measures that will be taken to safeguard the patient’s health and prevent recurrence of the error. Finally, offering a sincere apology, acknowledging responsibility, and providing emotional support are critical steps to uphold professional integrity, maintain trust, and promote healing in the physician-patient relationship.

This approach is consistent with the Medical Council of Thailand’s Regulations on the Maintenance of Medical Ethics issued under the Medical Profession Act, B.E. 2525 (1982) (Reprint 1996), which requires physicians to provide patients with accurate and sufficient information regarding their condition, treatment options, and associated risks, and prohibits false or misleading statements in communication and advertising [25]. It also aligns with the GMC’s “duty of candor” standard in the UK [27]. International experience also shows that open disclosure, when coupled with institutional commitment to a “just culture” (nonpunitive responses to error reporting), can improve patient outcomes, reduce malpractice claims, and preserve trust in the healthcare system.

Advertising and Communication

Outside direct patient encounters, deception can also occur in public communication and healthcare marketing. For example, during the COVID-19 pandemic, public communications in the UK and the USA frequently described vaccines as “safe and effective” without consistently acknowledging uncertainties, limitations of trial data, or rare adverse effects. While this messaging was intended to encourage uptake and reassure the public, its lack of qualification has been criticized as an oversimplification that risks misrepresentation. Such selective framing demonstrates how omissions, even when well intentioned, can undermine transparency, erode trust, and compromise ethical principles of honesty and justice by limiting truly informed decision-making [25].

Best practice requires that all health-related claims in advertising or promotion should be firmly grounded in evidence, ensuring that information provided to the public is accurate and trustworthy. Data must be presented with scientific accuracy and free from exaggeration or distortion so as not to mislead patients or create unrealistic expectations. Any uncertainty, limitations, or potential side effects associated with a treatment should be openly disclosed to support informed decision-making. Moreover, all promotional materials must align with established professional codes, such as the Thai Medical Council’s advertising guidelines [25], thereby upholding ethical standards and maintaining public trust in the medical profession.

This example demonstrates how deception extends beyond clinical care to professional self-promotion and institutional reputation management, making regulatory oversight essential to prevent exploitation of patients through misleading claims. Yet an additional question arises: who regulates the regulators? History shows that regulatory bodies themselves may be vulnerable to political influence, grouphink, or conflicts of interest. Ensuring accountability, therefore, requires mechanisms such as parliamentary or judicial scrutiny, independent review committees, external audits, transparent public reporting, and protections for whistleblowers. Such safeguards help maintain trust by ensuring that regulators are held to the same standards of honesty and transparency expected of clinicians and institutions.

Prognosis Discussion

Another critical domain is communication about prognosis. For instance, a patient with advanced cancer may be told an overly optimistic prognosis to “maintain hope,” while omitting crucial information about the likely disease course. While such actions may be motivated by compassion, they compromise patient autonomy and informed consent by depriving patients of the chance to prepare, make decisions about treatment preferences, or arrange for palliative care and family matters [26].

Best practice involves sharing prognosis information sensitively yet accurately, avoiding false reassurance that may compromise informed decision-making. Physicians must balance empathy with honesty, employing communication strategies such as the “ask-tell-ask” approach to assess the patient’s understanding, explore their preferences, and tailor the discussion accordingly. Alongside factual disclosure, it is essential to provide emotional support, helping patients and families cope with difficult news. In this context, hope can be reframed around achievable goals, such as maintaining quality of life, maximizing comfort, and preserving meaningful time with loved ones, rather than unrealistic expectations, thereby respecting autonomy while offering compassionate care.

This approach respects autonomy and aligns with both bioethical principles and professional standards such as the AMA Code of Medical Ethics [26], which emphasizes timely, truthful communication even in difficult circumstances.

Broader Implications

These case examples illustrate that deception and nondisclosure can take multiple forms: deception in medical practice can take multiple forms, each carrying significant ethical implications. Errors may be concealed, such as when medical mistakes are not disclosed to patients, thereby violating transparency and accountability in care. Evidence can also be distorted, as seen in misleading advertising or promotional communication that exaggerates treatment efficacy and undermines informed decision-making. Additionally, truth may be withheld in clinical interactions, for example, when prognosis discussions are framed with undue optimism or critical details are omitted, compromising patient autonomy and the validity of informed consent.

In each scenario, the consequences are profound, ranging from undermined autonomy to loss of trust in healthcare institutions. Conversely, applying best practices rooted in honesty, transparency, and empathy not only fulfills ethical and legal duties but also strengthens the therapeutic alliance and safeguards the reputation of the medical profession.

Communication strategies to minimize the risk of deception

Communication strategies such as involving a second clinician in difficult conversations, using structured protocols, and encouraging teach-back cannot eliminate deliberate deception, but they can minimize its likelihood and reduce unintentional lapses in honesty or transparency. Effective communication remains central to reducing the risk of deception and ensuring that patients receive accurate, comprehensible, and timely information. These strategies help safeguard patient autonomy, maintain trust, and limit misunderstanding while reinforcing ethical and professional standards.

Assessing Patient Needs and Readiness

Before delivering sensitive or complex information, clinicians should carefully evaluate the patient’s informational needs, emotional state, and readiness to receive news. This assessment should include considerations such as health literacy, cultural background, language preferences, and cognitive status. Tailoring the conversation to these factors helps ensure that the information is patient-centered and responsive, reducing the risk of confusion or distress.

Using Structured Protocols for Breaking Bad News

Evidence-based communication protocols, such as SPIKES (Setting, Perception, Invitation, Knowledge, Empathy, Summary/Strategy), provide a systematic framework for conveying difficult information. These structured approaches support clinicians in balancing honesty with compassion, pacing the disclosure appropriately, and creating opportunities for patients to ask questions and express emotions. Research has shown that the SPIKES model reduces patient distress, facilitates trust, and enhances shared decision-making by ensuring that both empathy and factual accuracy are maintained [28].

Using Plain Language and Avoiding Jargon

Medical jargon, technical terminology, and euphemisms can obscure meaning, leading to misinterpretation or a false sense of reassurance. To promote clarity, clinicians should adopt plain, straightforward language and confirm comprehension using strategies such as the “teach-back” method, where patients are invited to restate the information in their own words. This not only ensures understanding but also strengthens the patient’s role in their care.

Gradual Disclosure With Opportunities for Questions

Disclosing information gradually allows patients time to process and reflect, especially when the content is emotionally difficult or medically complex. Providing information in stages, interspersed with pauses for clarification, helps prevent information overload and encourages active patient participation. This incremental approach also fosters a sense of partnership between patient and provider, aligning the communication process with shared decision-making principles.

Documentation and Witnessing of Critical Communications

For significant or high-stakes conversations, such as error disclosure, informed consent for high-risk procedures, or prognosis discussions, documentation in the medical record is essential. Notes should detail the content of the discussion, its timing, and all participants present. In certain cases, having another healthcare professional, interpreter, or witness present not only enhances transparency but also provides additional emotional and practical support to the patient. This can also be fostered by inviting the patient to bring along a spouse, friend, or relative to the interview. The presence of a trusted companion can improve the patient’s retention and understanding, as it allows discussion afterwards and ensures that important details are not overlooked. This practice strengthens accountability and reinforces a culture of honesty and openness.

Institutional roles

Healthcare institutions play a pivotal role in fostering a culture of transparency, ethical integrity, and patient-centered communication. By implementing supportive systems and resources, institutions not only safeguard patients but also empower healthcare professionals to uphold high ethical standards even in difficult circumstances. Institutional responsibility ensures that honesty and openness are not left solely to individual discretion but embedded into the framework of healthcare delivery.

Establishing a Formal Error Disclosure Policy

Clear institutional policies on error disclosure provide a standardized approach to addressing medical mistakes, reducing ambiguity for clinicians and ensuring accountability. Such policies should include explicit definitions of what constitutes a reportable event, step-by-step procedures for disclosure, designated responsibilities for different team members, and required timelines for communication. Furthermore, documentation requirements must be outlined to ensure accuracy and traceability. When aligned with national laws, professional ethical codes, and patient rights charters, these policies reinforce consistency and fairness, ensuring that patients are informed in a timely, transparent, and respectful manner [29].

Training in Medical Ethics and Communication Skills

Institutions should provide ongoing training in medical ethics, patient-centered communication, and cultural competence. Regular workshops, role-play exercises, and simulation-based training allow healthcare professionals to practice scenarios such as breaking bad news, disclosing medical errors, or managing difficult family conversations. Interprofessional training, bringing together doctors, nurses, and allied health staff, can further strengthen team communication and reinforce shared responsibility for ethical practice. Evidence shows that systematic training in communication not only improves clinicians’ confidence but also enhances patient satisfaction, reduces complaints, and supports informed decision-making [29].

Providing Emotional and Professional Support for Staff

Adverse medical events impact not only patients but also the clinicians involved, who may suffer from emotional distress, guilt, loss of confidence, or fear of litigation. This “second victim” phenomenon has been increasingly recognized as a significant factor influencing professional performance and well-being. To mitigate these effects, institutions should establish structured support systems, including confidential counseling services, peer-support networks, and mentorship programs. Access to legal or risk management advice is also essential to address medicolegal concerns. By providing such resources, institutions help staff recover emotionally, maintain resilience, and continue to practice safely and ethically, while fostering a just culture where learning from mistakes is prioritized over blame [30].

While institutional policies and communication strategies are valuable in improving practice and minimizing inadvertent deception or nondisclosure, it is important to acknowledge their limitations. These measures cannot fully address intentional deception, whether enacted by an individual clinician, reinforced within an institution, or embedded in wider healthcare systems. Recognizing this limitation underscores the need for independent oversight, accountability mechanisms, and a culture that actively discourages concealment at all levels.

## Conclusions

Avoiding deception and nondisclosure is fundamental to maintaining trust in the physician-patient relationship, as it safeguards patient autonomy, promotes informed decision-making, and reinforces the ethical principles of honesty and beneficence. By fostering a culture of transparency, supported through structured training, clear institutional policies, and emotional support systems, healthcare providers can navigate difficult conversations with integrity while preserving therapeutic relationships. Ongoing research is needed to clarify the long-term impact of disclosure practices on patient trust, clinical outcomes, and litigation, ensuring that ethical commitments align with sustainable and effective healthcare delivery.
